# Polymorphisms in genes of melatonin biosynthesis and signaling support the light-at-night hypothesis for breast cancer

**DOI:** 10.1007/s10654-023-01048-7

**Published:** 2023-10-03

**Authors:** Katharina Wichert, Reiner Hoppe, Katja Ickstadt, Thomas Behrens, Stefan Winter, Robert Herold, Claudia Terschüren, Wing-Yee Lo, Pascal Guénel, Thérèse Truong, Manjeet K. Bolla, Qin Wang, Joe Dennis, Kyriaki Michailidou, Michael Lush, Irene L. Andrulis, Hermann Brenner, Jenny Chang-Claude, Angela Cox, Simon S. Cross, Kamila Czene, Mikael Eriksson, Jonine D. Figueroa, Montserrat García-Closas, Mark S. Goldberg, Ute Hamann, Wei He, Bernd Holleczek, John L. Hopper, Anna Jakubowska, Yon-Dschun Ko, Jan Lubiński, Anna Marie Mulligan, Nadia Obi, Valerie Rhenius, Mitul Shah, Xiao-Ou Shu, Jacques Simard, Melissa C. Southey, Wei Zheng, Alison M. Dunning, Paul D. P. Pharoah, Per Hall, Douglas F. Easton, Thomas Brüning, Hiltrud Brauch, Volker Harth, Sylvia Rabstein

**Affiliations:** 1 Institute for Prevention and Occupational Medicine of the German Social Accident Insurance, Institute of the Ruhr University Bochum (IPA), Bürkle-de-la-Camp-Platz 1, 44789 Bochum, Germany; 2 Dr. Margarete Fischer-Bosch-Institute of Clinical Pharmacology, Stuttgart, Germany; 3 University of Tübingen, Tübingen, Germany; 4 Department of Statistics, TU Dortmund University, Dortmund, Germany; 5 Institute for Occupational and Maritime Medicine Hamburg (ZfAM), University Medical Center Hamburg-Eppendorf (UKE), Hamburg, Germany; 6 Department of Clinical Pathology, University of Melbourne Centre for Cancer Research Victorian Comprehensive Cancer Centre Melbourne, Melbourne, VIC Australia; 7 Team “Exposome and Heredity”, CESP, Gustave Roussy, INSERM, University Paris-Saclay, UVSQ, Villejuif, France; 8 Centre for Cancer Genetic Epidemiology, Department of Public Health and Primary Care, University of Cambridge, Cambridge, UK; 9 Biostatistics Unit, The Cyprus Institute of Neurology & Genetics, Nicosia, Cyprus; 10 Cyprus School of Molecular Medicine, The Cyprus Institute of Neurology & Genetics, Nicosia, Cyprus; 11 Fred A. Litwin Center for Cancer Genetics, Lunenfeld-Tanenbaum Research Institute of Mount Sinai Hospital, Toronto, ON Canada; 12 Department of Molecular Genetics, University of Toronto, Toronto, ON Canada; 13 Division of Clinical Epidemiology and Aging Research, German Cancer Research Center (DKFZ), Heidelberg, Germany; 14 Division of Preventive Oncology, German Cancer Research Center (DKFZ) and National Center for Tumor Diseases (NCT), Heidelberg, Germany; 15 German Cancer Consortium (DKTK), German Cancer Research Center (DKFZ), Heidelberg, Germany; 16 Division of Cancer Epidemiology, German Cancer Research Center (DKFZ), Heidelberg, Germany; 17 Cancer Epidemiology Group, University Cancer Center Hamburg (UCCH), University Medical Center Hamburg-Eppendorf, Hamburg, Germany; 18 Sheffield Institute for Nucleic Acids (SInFoNiA), Department of Oncology and Metabolism, University of Sheffield, Sheffield, UK; 19 Academic Unit of Pathology, Department of Neuroscience, University of Sheffield, Sheffield, UK; 20 Department of Medical Epidemiology and Biostatistics, Karolinska Institutet, Stockholm, Sweden; 21 Usher Institute of Population Health Sciences and Informatics, The University of Edinburgh, Edinburgh, UK; 22 Cancer Research UK Edinburgh Centre, The University of Edinburgh, Edinburgh, UK; 23 Division of Cancer Epidemiology and Genetics, Department of Health and Human Services, National Cancer Institute, National Institutes of Health, Bethesda, MD USA; 24 Department of Medicine, McGill University, Montréal, QC Canada; 25 Division of Clinical Epidemiology, Royal Victoria Hospital, McGill University, Montréal, QC Canada; 26 Molecular Genetics of Breast Cancer, German Cancer Research Center (DKFZ), Heidelberg, Germany; 27 Saarland Cancer Registry, Saarbrücken, Germany; 28 Centre for Epidemiology and Biostatistics, Melbourne School of Population and Global Health, The University of Melbourne, Melbourne, VIC Australia; 29 Department of Genetics and Pathology, International Hereditary Cancer Center, Pomeranian Medical University, Szczecin, Poland; 30 Independent Laboratory of Molecular Biology and Genetic Diagnostics, Pomeranian Medical University, Szczecin, Poland; 31 Department of Internal Medicine, Johanniter GmbH Bonn, Johanniter Krankenhaus, Bonn, Germany; 32 Department of Laboratory Medicine and Pathobiology, University of Toronto, Toronto, ON Canada; 33 Laboratory Medicine Program, University Health Network, Toronto, ON Canada; 34 Institute for Medical Biometry and Epidemiology, University Medical Center Hamburg-Eppendorf, Hamburg, Germany; 35 Centre for Cancer Genetic Epidemiology, Department of Oncology, University of Cambridge, Cambridge, UK; 36 Division of Epidemiology, Department of Medicine, Vanderbilt Epidemiology Center, Vanderbilt-Ingram Cancer Center, Vanderbilt University School of Medicine, Nashville, TN USA; 37 Genomics Center, Centre Hospitalier Universitaire de Québec – Université Laval Research Center, Québec City, QC Canada; 38 Precision Medicine, School of Clinical Sciences at Monash Health, Monash University, Clayton, VIC Australia; 39 Department of Clinical Pathology, The University of Melbourne, Melbourne, VIC Australia; 40 Cancer Epidemiology Division, Cancer Council Victoria, Melbourne, VIC Australia; 41 Department of Oncology, Södersjukhuset, Stockholm, Sweden; 42 iFIT-Cluster of Excellence, University of Tübingen, Tübingen, Germany; 43 German Cancer Consortium (DKTK) and German Cancer Research Center (DKFZ), Partner Site Tübingen, Tübingen, Germany

**Keywords:** *TPH2*, *MAPK8*, Serotonin biosynthesis, Circadian rhythm, Shift work

## Abstract

Light-at-night triggers the decline of pineal gland melatonin biosynthesis and secretion and is an IARC-classified probable breast-cancer risk factor. We applied a large-scale molecular epidemiology approach to shed light on the putative role of melatonin in breast cancer. We investigated associations between breast-cancer risk and polymorphisms at genes of melatonin biosynthesis/signaling using a study population of 44,405 women from the Breast Cancer Association Consortium (22,992 cases, 21,413 population-based controls). Genotype data of 97 candidate single nucleotide polymorphisms (SNPs) at 18 defined gene regions were investigated for breast-cancer risk effects. We calculated adjusted odds ratios (ORs) and 95% confidence intervals (CI) by logistic regression for the main-effect analysis as well as stratified analyses by estrogen- and progesterone-receptor (ER, PR) status. SNP-SNP interactions were analyzed via a two-step procedure based on logic regression. The Bayesian false-discovery probability (BFDP) was used for all analyses to account for multiple testing. Noteworthy associations (BFDP < 0.8) included 10 linked SNPs in *tryptophan hydroxylase 2* (*TPH2*) (e.g. rs1386492: OR = 1.07, 95% CI 1.02–1.12), and a SNP in the *mitogen-activated protein kinase 8* (*MAPK8*) (rs10857561: OR = 1.11, 95% CI 1.04–1.18). The SNP-SNP interaction analysis revealed noteworthy interaction terms with *TPH2-* and MAPK-related SNPs (e.g. rs1386483_*R*_ ∧ rs1473473_*D*_ ∧ rs3729931_*D*_: OR = 1.20, 95% CI 1.09–1.32). In line with the light-at-night hypothesis that links shift work with elevated breast-cancer risks our results point to SNPs in *TPH2* and MAPK-genes that may impact the intricate network of circadian regulation.

**Supplementary Information:**

The online version contains supplementary material available at 10.1007/s10654-023-01048-7.

## Introduction

Breast cancer is the most common cancer and the leading cause of cancer death for women worldwide with a higher incidence among women in developed countries [1]. Besides several reproductive and lifestyle-associated risk factors [2], exposure to light-at-night has been suggested to promote breast cancer [3]. In 2007, the International Agency for Research on Cancer (IARC) classified shift work that includes circadian disruption as probably carcinogenic to humans (group 2A) [4]. In their 2019 re-evaluation the IARC confirmed and specified this classification to night-shift work. Studies on the effects of light in animal bioassays were key to this evaluation [5]. Although risk estimates between epidemiological studies vary due to different exposure assessments and study populations, a large pooled analysis of case–control studies confirmed the association between a high number of night shifts and breast cancer [6].

The circadian system is orchestrated by a multisynaptic pathway that is governed by a master clock, the suprachiasmatic nucleus (SCN), located in the hypothalamus. Following photic input, the pathway is set into operation via the retino-hypothalamic tract by intrinsically photosensitive retinal ganglion cells. The light signal is directly projected into the SCN to finally synapse with the pineal gland via complex networks including the sympathetic nervous system, superior cervical ganglions as well as other participating hypothalamic areas (paraventricular nucleus, PVN) [7, 8]. In the circadian clock, mitogen-activated protein kinase (MAPK) pathways function both as input pathways to maintain or reset the oscillator to 24 h environmental cycles, and output pathways that connect the timekeeping oscillator through control of the expression of a large number of functionally related genes [9]. Several variants in circadian genes have been linked to general breast-cancer susceptibility [10, 11].

Melatonin (N-acetyl-5-methoxytryptamine) is the key-player for this synchronization of bodily circadian rhythms. Its biosynthesis follows a multistep process starting with the hydroxylation of the precursor molecule L-tryptophan catalyzed by tryptophan hydroxylase (TPH). Decarboxylation of 5-hydroxy-L-tryptophan by L-aromatic amino acid decarboxylase (AADC) results in the neurotransmitter serotonin, the acetylation of which by aralkylamine *N*-acetyltransferase (AANAT) and methylation by *N*-acetylserotonin O-methyltransferase (ASMT, alias HIOMT) finally yields melatonin [12]. During darkness, AANAT activity increases via phosphorylation thereby blocking its proteasomal proteolysis, and its high affinity to serotonin leads to a strong increase in melatonin production [12].

Melatonin is mainly secreted from the pineal gland upon photic neural input, but also produced by other ocular tissues such as photoreceptors and ciliary body epithelium, albeit to a lesser extent, as well as other bodily tissues [13, 14]. With its secretion being affected by the light–dark cycle, melatonin synchronizes bodily circadian rhythms relevant to many endogenous processes including the production of sex hormones [15, 16]. The desynchronization of SCN activity either by day length or timing/phasing of light exposure consequently affects the production of melatonin by the pineal gland and is referred to as circadian disruption [3, 17].

The light-at-night-associated breast-cancer risk has been attributed to a reduced nocturnal biosynthesis and lower secretion of melatonin [3, 17]. In particular, an increased risk for hormone-sensitive breast cancer has been mechanistically accredited to a modified crosstalk between melatonin-receptor and estrogen-receptor pathways triggered upon reduced melatonin and modulated estrogen exposure [18]. Here we investigated the putative contribution of genetic polymorphisms of key enzymes of melatonin biosynthesis and signaling to the risk of developing breast cancer, and highlight a cooperative role in favor of this risk based on a large international association study of more than 44,000 breast-cancer cases and controls.

## Material and methods

### Study population

**Fig. 1:**
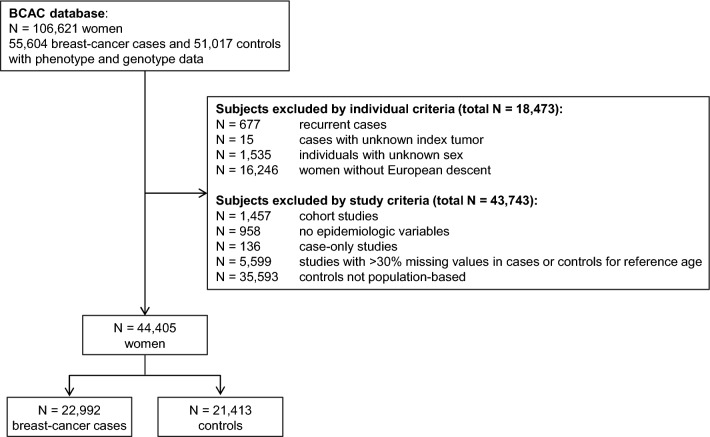
Flow chart of BCAC data set for the inclusion of case-control studies in the analysis, detailed information on individual studies is provided in Suppl. table S1

We screened 106,621 breast-cancer cases and control subjects with available pheno- and genotype data deposited in the database of the Breast Cancer Association Consortium (BCAC) [19, 20] at the University of Cambridge. Exclusion criteria at the study and individual level are specified in Fig. 1. Studies were not included if reference age (age at diagnosis for cases, age at interview for controls) was missing in > 30% of the study participants, relevant epidemiological variables were not recorded, or controls were not population-based or a case-only design was used. All subjects had to be women as well as of European descent, and cases were required to have a diagnosis of primary breast cancer. Based on these criteria, 44,405 eligible women (22,992 cases and 21,413 controls) from 14 population-based case–control studies were included in the analysis. Individual study descriptions are given in Supplementary table S1. All studies were approved by local ethics committees and all participants gave informed consent.

### Polymorphisms and genotype data

We focused on 97 single nucleotide polymorphisms (SNPs) at 18 genes including melatonin biosynthesis (e.g. *TPH1* and *TPH2*), melatonin receptors (*MTNR1A* and *MTNR1B)* as well as various MAP kinases (e.g. *MAP2K1*, *MAP2K2*, *MAPK1*, *MAPK8*). All 97 SNPs are listed in Supplementary table S2 together with their characteristics in the study population. Corresponding genotypes were retrieved from the BCAC database Cambridge. They were previously generated within the framework of the Collaborative Oncological Gene-environment Study (COGS) using a custom Illumina iSelect array with 211,155 SNPs as described elsewhere [19]. For the SNP selection, all available SNPs on the array at the aforementioned genes were considered.

### Statistical analysis

#### Quality criteria

We checked for Hardy–Weinberg-Equilibrium (HWE) by Χ^2^-tests and analyzed the heterogeneity between studies by calculating Cochran’s Q for the heterozygous and homozygous rare genotypes for each SNP (Suppl. table S2). To consider multiple testing, we used 0.05 divided by the number of analyzed SNPs as threshold for *p* values.

#### Main-effect analysis and confounder selection

For the main-effect analysis of each SNP, we estimated odds ratios (ORs) and 95% confidence intervals (CIs) for each SNP assuming different genetic models (dominant, recessive, and additive model). All models were adjusted for reference age, study, and a set of eight principal components to consider a possible population stratification effect. Furthermore, we adjusted these models for parity (nulliparous/1+ full term pregnancies/unknown), breast-feeding status (never/ever/unknown), smoking status (never/ever/unknown), and current use of estrogen-progesterone combined menopausal hormone therapy (MHT) (no/yes/unknown). Regarding menopausal hormone therapy, current use was defined as ‘use at reference date or within six months prior to the reference date’. Missing values in categorical covariates were coded as ‘unknown’. In a sensitivity analysis, we additionally adjusted the models for menopausal status (pre- and peri-menopausal/post-menopausal/unknown). Post-menopausal was defined as ‘last menstruation more than 12 months before the reference date’. We also calculated ORs separately for estrogen-receptor (ER) positive/negative (ER±) and progesterone-receptor (PR) positive/negative (PR±) cases.

#### Interaction analyses with logic regression

To analyze interactions between SNPs, we used a two-staged procedure based on logic regression models [21]. In short, a logic regression model is a so-called logic tree embedded in a generalized linear model. The logic tree consists of binary covariates linked by logic expressions with the AND-expression (conjunction) representing interactions (notation of interactions: A $\wedge$ B (A and B); C $\wedge$ !D (C and not D)). An optimization algorithm is used to select interactions for the logic tree. Here, we used the logit as link function of the framing generalized linear model with the case–control status as outcome and the simulated annealing algorithm to select interactions for the logic tree as independent variable. To express SNP-SNP interactions in logic regression models, SNP coding in the dominant and recessive genetic model was required [21].

In the first stage of our procedure, we selected interactions for the logic tree by using the logic Feature Selection (logicFS) algorithm to avoid overfitting [21]. Here, we used logicFS to fit 100 logic regression models from bootstrap samples and to calculate a variable importance measure for the multiple tree approach based on the number of correctly classified out-of-bag observations for each bootstrap sample for every interaction consisting of up to six terms included in these models [21]. We ran the algorithm three times with a different random seed and selected the 20 most important interactions each. In the second stage, we fitted individual adjusted logistic regression models with the selected terms.

To account for multiple testing and an increased type I error rate, we calculated the Bayesian false-discovery probability (BFDP) for SNPs/interaction terms with a *p* value < 0.05 in the adjusted models, assuming a four-fold cost of a false non-discovery compared to a false discovery as suggested by Wakefield [22]. Effects with BFDP < 0.8 are termed noteworthy. We calculated the BFDP for three different prior probabilities (0.1, 0.05, 0.01) for a true association and the OR corresponding to the 97.5% quantile of the prior OR was set to 1.2 for positive associations and to 0.83 for negative associations. Linkage disequilibrium was checked for noteworthy SNPs.

The statistical software R, version 3.4.2, was used for all calculations [23]. The R-packages ‘logicFS’ and ‘LogicReg’ were used for the interaction analysis [24, 25]. All statistical models were fitted as complete-case analyses, including the category ‘unknown’ for missing values in categorical variables, therefore the number of individuals available for calculations varied respectively. This also accounts for slight differences in ORs between the main-effect analysis and the interaction analysis, when an interaction term consists of only one SNP.

## Results

The study population of 44,405 women was contributed by 14 case–control studies (Suppl. table S1) of which the smallest study comprised 243 women (NBHS) and the largest 16,746 women (SEARCH). Among the eligible 22,992 cases and 21,413 controls, the mean reference age was 56 years for cases and 57 years for controls with a standard deviation of 10 and 9 years, respectively. Most women had at least one full term pregnancy (76%) and nearly 50% had ever breastfed (Table 1).

**Table 1:** Characteristics of the study population composed of 14 eligible case-control studies from the BCAC data base

	Total		ABCFS		CECILE		ESTHER		GENICA		MARIE		MTLGEBCS		NBHS		OFBCR	
	Cases	Controls	Cases	Controls	Cases	Controls	Cases	Controls	Cases	Controls	Cases	Controls	Cases	Controls	Cases	Controls	Cases	Controls
N	22,992	21,413	770	492	1,016	994	475	502	465	427	1,816	1,778	408	360	125	118	1,056	495
Reference age [years] (mean (SD))	56 (10)	57 (9)	40 (7)	42 (9)	54 (11)	55 (11)	61 (9)	62 (7)	57 (11)	57 (12)	62 (6)	62 (6)	62 (6)	61 (6)	53 (11)	53 (11)	53 (10)	52 (9)
Parity (n)																		
Nulliparous 1+ full term pregnancies Unknown	2,962 16,580 3,450	2,094 17,135 2,184	185 585 0	119 373 0	109 907 0	66 928 0	53 421 1	44 451 7	80 385 0	86 341 0	292 1,524 0	268 1,510 0	0 0 408	0 0 360	0 0 125	0 0 118	299 757 0	77 418 0
Ever breastfed (n)																		
No Yes Unknown	5,469 11,536 5,987	4,214 10,320 6,879	278 492 0	169 323 0	410 466 140	349 500 145	53 269 153	50 301 151	215 247 3	184 243 0	661 1,155 0	601 1,177 0	0 0 408	0 0 360	0 0 125	0 0 118	548 508 0	203 292 0
Family history of breast cancer (n)																		
No Yes Unknown	16,581 3,920 2,491	14,993 1,864 4,556	631 139 0	455 37 0	782 178 56	837 97 60	351 74 50	371 43 88	404 61 0	397 30 0	1,470 303 43	1,517 213 48	305 103 0	306 54 0	100 25 0	93 25 0	626 429 1	433 54 8
Ever smoking (n)																		
No Yes Unknown	6,279 6,601 10,112	8,202 7,408 5,803	348 421 1	235 257 0	617 399 0	601 393 0	297 175 3	325 166 11	270 195 0	225 202 0	989 827 0	950 827 1	0 0 408	0 0 360	0 0 125	0 0 118	424 479 153	239 256 0
Menopausal status (n)																		
Pre-/peri-menopausal Post-menopausal Unknown	5,851 12,933 4,208	5,526 12,167 3,720	534 139 97	220 129 143	375 577 64	341 593 60	50 412 13	25 454 23	128 331 6	118 304 5	221 1,595 0	176 1,602 0	0 0 408	0 0 360	10 26 89	17 23 78	189 714 153	217 278 0
Current use of estrogen-progesterone combined therapy (n)																		
No Yes Unknown	10,725 1,168 11,099	10,788 739 9,886	707 0 63	416 0 76	875 76 65	853 63 78	190 0 285	220 0 282	419 45 1	386 41 0	1,306 505 5	1,397 373 8	0 0 408	0 0 360	20 0 105	23 0 95	543 0 513	282 0 213
Tumor: ER status (n)																		
Positive (ER+) Negative (ER−) Unknown	14,724 3,516 4,752		447 254 69		805 141 70		302 98 75		336 119 10		1,347 400 69		353 53 2		0 125 0		595 250 211	
Tumor: PR status (n)																		
Positive (PR+) Negative (PR−) Unknown	10,016 4,768 8,208		500 201 69		665 271 80		260 135 80		313 142 10		1,141 605 70		303 102 3		0 125 0		504 322 230	

	PBCS		pKARMA		SASBAC		SBCS		SEARCH		SZBCS	
	Cases	Controls	Cases	Controls	Cases	Controls	Cases	Controls	Cases	Controls	Cases	Controls
N	519	424	5,335	5,226	1,148	1,378	818	840	8,682	8,064	359	315
Reference age [years] (mean (SD))	56 (10)	56 (10)	58 (10)	53 (10)	63 (6)	63 (6)	59 (12)	57 (6)	54 (9)	58 (9)	56 (11)	57 (10)
Parity (n)												
Nulliparous 1+ full term pregnancies Unknown	85 434 0	42 382 0	817 4,470 48	196 4,553 477	164 984 0	133 1,245 0	123 695 0	99 741 0	755 5,418 2,509	964 6,193 907	0 0 359	0 0 315
Ever breastfed (n)												
No Yes Unknown	174 345 0	107 317 0	950 4,207 178	556 4,102 568	206 827 115	180 961 237	202 192 424	179 183 478	1,772 2,828 4,082	1,636 1,921 4,507	0 0 359	0 0 315
Family history of breast cancer (n)												
No Yes Unknown	473 46 0	395 29 0	4,189 979 167	4,196 560 470	0 176 972	0 116 1,262	701 117 0	758 82 0	6,363 1,251 1,068	5,218 524 2,322	186 39 134	17 0 298
Ever smoking (n)												
No Yes Unknown	222 297 0	203 220 1	2,186 3,095 54	2,401 2,816 9	650 498 0	793 585 0	182 109 527	440 400 0	36 29 8617	1,790 1,286 4,988	58 77 224	0 0 315
Menopausal status (n)												
Pre-/peri-menopausal Post-menopausal unknown	128 391 0	122 302 0	1,284 3,918 133	2,397 2,645 184	1 1,147 0	5 1,373 0	268 548 2	268 572 0	2,639 3,105 2,938	1,620 3,892 2,552	24 30 305	0 0 315
Current use of estrogen-progesterone combined therapy (n)												
No Yes Unknown	438 54 27	374 25 25	4,639 270 426	4,446 65 715	909 218 21	1,166 172 40	634 0 184	469 0 371	45 0 8,637	756 0 7,308	0 0 359	0 0 315
Tumor: ER status (n)												
Positive (ER+) Negative (ER−) Unknown	519 0 0		3,751 716 868		663 144 341		375 106 337		4,996 1,034 2,652		235 76 48	
Tumor: PR status (n)												
Positive (PR+) Negative (PR−) Unknown	358 160 1		3,047 1,338 950		559 227 362		125 84 609		2,179 1,003 5,500		62 53 244	

*SD* Standard deviation, *ER* Estrogen receptor, *PR* Progesterone receptor, *ER* ± Estrogen-receptor status positive/negative, *PR* ± Progesterone-receptor status positive/negative

Most of the 97 analyzed SNPs had very few missing values, with a maximum of 4% for rs10217741 (*RORB*). The minor allele frequency (MAF) in controls ranged from 2–49%. Detailed information for all SNPs is provided in Supplementary table S2. HWE was not met for rs10765576 (*MTNR1B*) and rs14303 (*MAP2K1*), and therefore, these polymorphisms were not followed up further.

### Main-effect analysis

Noteworthy associations (BFDP < 0.8) between individual SNPs and breast-cancer risk have been identified particularly for *TPH2* intronic polymorphisms*.* ORs (95% CIs) of individual SNPs with a *p* value < 0.05 and respective BFDPs with different priors (0.1, 0.05, and 0.01) for an effect in the adjusted dominant or recessive model are given in Table 2. ORs and CIs for all SNPs and models are given in Supplementary table S3. The largest OR (adjusted for study, reference age, parity, breast feeding, smoking status, current intake of estrogen-progesterone MHT, and principle components) was observed for recessive rs10857561 (*MAPK8*, OR = 1.11, 95% CI 1.04–1.18, BFDP < 0.8 at prior 0.01). Increasing the prior to 0.05 also revealed eight linked noteworthy dominant *TPH2* SNPs (rs7300641, rs1386492, rs1473473, rs4760751, rs1487276, rs1386489, rs1487281, rs7299582) with similar adjusted ORs around 1.07 (95% CIs 1.02–1.12) in the dominant model. At a prior of 0.1, protective effects were revealed for two *TPH2* SNPs in the recessive model (rs17110627, OR = 0.91, 95% CI 0.83–0.99; and rs2129575, OR = 0.91, 95% CI 0.83–0.99), as well as recessive rs13515 (*MAPK1,* OR = 0.89, 95% CI 0.79–0.99). Recessive rs7075976 (*MAPK8*, OR = 1.06, 95% CI 1.01–1.12) was also noteworthy. The additive model showed similar results (Suppl. table S3). A sensitivity analysis with additional adjustment for menopausal status did not change the results. When we compared our results with those obtained in the meta GWAS analysis, none of the noteworthy SNP associations reported here were identified at the genome wide association level (5E-08) [20, 26, 27].

**Table 2:** Breast-cancer risk associations and Bayesian false-discovery probability (BFDP, bold text indicates BFDP < 0.8) of individual SNPs with *p* values < 0.05 in adjusted dominant and recessive logistic regression models*, sorted by OR^b^

SNP	Nearest gene	Chromosome, position (GRCh38.p13), and referent/variant alleles	OR^a^ (95% CI)	*p* value OR^a^	OR^b^ (95% CI)	*p* value OR^b^	BFDP for OR^b^ Prior probability for effect (β ≠ 0)		
							0.1	0.05	0.01
*dominant*									
rs7300641^#^	*TPH2*	chr12:71,974,791: C:A	1.046 (1.005–1.089)	0.0290	1.071 (1.025–1.119)	0.0023	**0.3113**	**0.4883**	0.8326
rs1386492^#^	*TPH2*	chr12:71,968,485: A:G	1.046 (1.005–1.089)	0.0282	1.070 (1.024–1.118)	0.0026	**0.3393**	**0.5202**	0.8496
rs1473473^$^	*TPH2*	chr12:72,010,598: A:G	1.050 (1.009–1.094)	0.0168	1.066 (1.020–1.114)	0.0042	**0.4562**	**0.6391**	0.9022
rs4760751^§^	*TPH2*	chr12:71,984,138: G:A	1.040 (0.997–1.084)	0.0698	1.066 (1.018–1.116)	0.0064	**0.5240**	**0.6992**	0.9237
rs1487276^$^	*TPH2*	chr12:72,011,279: G:A	1.050 (1.008–1.093)	0.0184	1.065 (1.019–1.113)	0.0049	**0.4855**	**0.6658**	0.9121
rs1386489^§^	*TPH2*	chr12:71,955,510: A:G	1.040 (0.998–1.085)	0.0635	1.065 (1.017–1.115)	0.0072	**0.5509**	**0.7214**	0.9310
rs1487281^§^	*TPH2*	chr12:71,986,242: A:C	1.036 (0.994–1.081)	0.0950	1.062 (1.014–1.112)	0.0101	**0.6267**	**0.7799**	0.9486
rs7299582^§^	*TPH2*	chr12:71,962,534: A:G	1.037 (0.994–1.081)	0.0915	1.062 (1.014–1.112)	0.0101	**0.6267**	**0.7799**	0.9486
rs17110627	*TPH2*	chr12:71,996,615: G:A	0.967 (0.931–1.005)	0.0844	0.958 (0.919–0.998)	0.0412	0.8461	0.9207	0.9837
*recessive*									
rs10857561	*MAPK8*	chr10:48,400,595: G:A	1.118 (1.056–1.184)	0.0001	1.106 (1.039–1.177)	0.0016	**0.2344**	**0.3926**	**0.7710**
rs7075976	*MAPK8*	chr10:48,406,234: A:G	1.064 (1.015–1.116)	0.0097	1.064 (1.010–1.120)	0.0193	**0.7110**	0.8385	0.9644
rs2129575	*TPH2*	chr12:71,946,293: C:A	0.932 (0.859–1.012)	0.0932	0.907 (0.830–0.991)	0.0314	**0.7572**	0.8682	0.9717
rs17110627	*TPH2*	chr12:71,996,615: G:A	0.940 (0.868–1.018)	0.1268	0.905 (0.829–0.987)	0.0235	**0.7246**	0.8475	0.9666
rs13515	*MAPK1*	chr22:21,761,597: G:A	0.930 (0.840–1.031)	0.1678	0.886 (0.792–0.991)	0.0341	**0.7713**	0.8769	0.9738

*BFDP* Bayesian false-discovery probability, *OR* Odds ratio, *CI* Confidence interval

Chromosome and position (GRCh38.p13): For example, chr9:91,426,574: A:G indicates chromosome 9, base pair location 91,426,574, referent allele A, variant allele G;

^a^Adjusted for study, reference age and eight principal components

^b^Adjusted for study, reference age, parity, breast feeding, smoking status, current intake of estrogen-progesterone MHT, and eight principal components

*Supplementary table S3 provides an overview of all models for the main analysis

^#^, ^$^, ^§^, ^%^, and ^†^ mark SNPs that are pairwise linked (r^2^ > 0.95) in controls: ^#^ rs7300641 and rs1386492; ^$^ rs1473473 and rs1487276; ^§^ rs4760751, rs1386489, rs1487281, and rs7299582

### Analysis by tumor hormone-receptor status

Tumor hormone-receptor status was ER-positive (ER+) for 14,724 patients and ER-negative (ER−) for 3516 patients, as well as PR-positive (PR+) for 10,016 patients and PR-negative (PR−) for 4,768 patients (Table 1). Noteworthy breast-cancer risk associations (BFDP < 0.8) in the ER+ , ER−, PR+ , and PR− subgroups for the dominant and recessive model are listed in Table 3. Results for all SNPs and models are given in Supplementary table S4. None of the four tumor-hormone-receptor subgroups showed noteworthy associations at a prior of 0.01. At a prior of 0.05, six SNPs showed noteworthy associations. These comprised *MAPK8* SNP rs10857561 in the ER+ subgroup (OR = 1.10, 95% CI 1.02–1.18; recessive model) and two tightly linked *TPH2* SNPs rs7300641 and rs1386492 in the PR+ group (both: OR = 1.07, 95% CI 1.02–1.13; dominant model). In the ER− subgroup the two linked *TPH2* SNPs rs1473473 and rs1487276 (both: OR = 1.12, 95% CI 1.04–1.22; dominant model) and the *RORA* SNP rs17237290 (OR = 0.85, 95% CI 0.73–0.97; dominant model) showed noteworthy associations, while none of the associations in the PR− subgroup were noteworthy at prior 0.05. Besides rs17237290, the variants mentioned above were also noteworthy in the main analysis at prior 0.05 under the identical genetic models. At a prior of 0.1, in total 25 noteworthy associations of 19 SNPs were observed for all four breast cancer subtypes.

**Table 3:** Breast-cancer risk associations and Bayesian false-discovery probability (BFDP, bold text indicates BFDP < 0.8) of individual SNPs with *p* values < 0.05 in adjusted logistic regression models for dominant or recessive models* stratified by tumor hormone-receptor status, each subgroup sorted by OR^b^

	SNP	Nearest gene	Chromosome, position (GRCh38.p13), and referent/variant alleles	OR^a^ (95% CI)	*p* value OR^a^	OR^b^ (95% CI)	*p* value OR^b^	BFDP for OR^b^ Prior probability for effect (β ≠ 0)		
								**0.1**	**0.05**	**0.01**
ER+	*dominant*									
	rs7300641	*TPH2*	chr12:71,974,791: C:A	1.032 (0.986–1.081)	0.1704	1.063 (1.012–1.117)	0.0147	**0.6935**	0.8269	0.9614
	rs1386492	*TPH2*	chr12:71,968,485: A:G	1.033 (0.987–1.081)	0.1685	1.063 (1.011–1.117)	0.0159	**0.6934**	0.8269	0.9614
	rs1473473	*TPH2*	chr12:72,010,598: A:G	1.034 (0.987–1.082)	0.1564	1.054 (1.003–1.107)	0.0375	0.8156	0.9033	0.9799
	rs1487276	*TPH2*	chr12:72,011,279: G:A	1.033 (0.987–1.081)	0.1617	1.053 (1.002–1.106)	0.0414	0.8268	0.9097	0.9813
	*recessive*									
	rs7032571	*NFIL3*	chr9:91,428,391: A:G	1.183 (1.043–1.342)	0.0090	1.150 (1.003–1.317)	0.0446	0.8021	0.8954	0.9781
	rs10857561	*MAPK8*	chr10:48,400,595: G:A	1.107 (1.037–1.181)	0.0021	1.097 (1.023–1.177)	0.0095	**0.5810**	**0.7453**	0.9385
	rs1470747	*DDC*	chr7:50,559,312: G:A	0.917 (0.838–1.004)	0.0602	0.902 (0.819–0.994)	0.0373	**0.7798**	0.8820	0.9750
PR+	*dominant*									
	rs7300641	*TPH2*	chr12:71,974,791: C:A	1.053 (1.000–1.110)	0.0520	1.074 (1.016–1.134)	0.0109	**0.6005**	**0.7604**	0.9430
	rs1386492	*TPH2*	chr12:71,968,485: A:G	1.053 (1.000–1.110)	0.0518	1.073 (1.016–1.133)	0.0117	**0.6205**	**0.7754**	0.9473
	rs4760751	*TPH2*	chr12:71,984,138: G:A	1.047 (0.991–1.106)	0.0993	1.063 (1.004–1.126)	0.0354	0.8070	0.8983	0.9787
	rs1386489	*TPH2*	chr12:71,955,510: A:G	1.047 (0.992–1.106)	0.0970	1.062 (1.003–1.125)	0.0381	0.8167	0.9039	0.9800
	rs1473473	*TPH2*	chr12:72,010,598: A:G	1.052 (0.998–1.108)	0.0596	1.060 (1.002–1.120)	0.0377	0.8126	0.9015	0.9795
	rs1487276	*TPH2*	chr12:72,011,279: G:A	1.052 (0.998–1.108)	0.0592	1.059 (1.003–1.119)	0.0388	0.8225	0.9072	0.9808
	rs7299582	*TPH2*	chr12:71,962,534: A:G	1.043 (0.988–1.102)	0.1272	1.059 (1.001–1.122)	0.0472	0.8424	0.9186	0.9833
	*recessive*									
	rs7032571	*NFIL3*	chr9:91,428,391: A:G	1.216 (1.054–1.402)	0.0072	1.190 (1.025–1.381)	0.0224	**0.7467**	0.8616	0.9701
	rs10857561	*MAPK8*	chr10:48,400,595: G:A	1.090 (1.012–1.175)	0.0238	1.092 (1.010–1.181)	0.0276	**0.7466**	0.8615	0.9701
ER−	*dominant*									
	rs1473473	*TPH2*	chr12:72,010,598: A:G	1.109 (1.025–1.198)	0.0095	1.124 (1.036–1.220)	0.0051	**0.4596**	**0.6423**	0.9034
	rs1487276	*TPH2*	chr12:72,011,279: G:A	1.109 (1.026–1.198)	0.0095	1.124 (1.036–1.220)	0.0052	**0.4596**	**0.6423**	0.9034
	rs4760751	*TPH2*	chr12:71,984,138: G:A	1.075 (0.991–1.165)	0.0823	1.099 (1.009–1.197)	0.0294	**0.7560**	0.8674	0.9715
	rs1386489	*TPH2*	chr12:71,955,510: A:G	1.071 (0.988–1.161)	0.0975	1.095 (1.005–1.192)	0.0375	**0.7782**	0.8810	0.9747
	rs7299582	*TPH2*	chr12:71,962,534: A:G	1.067 (0.984–1.158)	0.1159	1.091 (1.002–1.188)	0.0460	0.8035	0.8962	0.9783
	rs17237290	*RORA*	chr15:60,579,671: A:G	0.880 (0.769–1.008)	0.0643	0.845 (0.734–0.972)	0.0187	**0.6030**	**0.7623**	0.9435
	rs16942767	*RORA*	chr15:60,569,169: G:A	0.873 (0.760–1.002)	0.0533	0.831 (0.720–0.960)	0.0121	**0.6749**	0.8142	0.9581
	rs16942772	*RORA*	chr15:60,574,930: C:A	0.869 (0.757–0.997)	0.0459	0.829 (0.718–0.958)	0.0109	**0.6661**	0.8081	0.9564
	*recessive*									
	rs12229394	*TPH2*	chr12:71,999,134: G:A	0.868 (0.747–1.008)	0.0627	0.836 (0.715–0.978)	0.0251	**0.7660**	0.8736	0.9730
	rs17110627	*TPH2*	chr12:71,996,615: G:A	0.847 (0.718–0.998)	0.0477	0.814 (0.685–0.967)	0.0191	**0.7547**	0.8666	0.9713
PR−	*dominant*									
	rs2289858	*MAP2K2*	chr19:4,102,625: A:G	1.096 (0.999–1.203)	0.0518	1.110 (1.009–1.221)	0.0328	**0.7610**	0.8705	0.9722
	rs1473473	*TPH2*	chr12:72,010,598: A:G	1.066 (0.995–1.142)	0.0674	1.083 (1.008–1.162)	0.0285	**0.7454**	0.8608	0.9699
	rs1487276	*TPH2*	chr12:72,011,279: G:A	1.065 (0.994–1.141)	0.0725	1.081 (1.007–1.160)	0.0316	**0.7648**	0.8729	0.9728
	rs1549854	*MAP2K1*	chr15:66,404,397: C:A	0.915 (0.851–0.985)	0.0176	0.918 (0.851–0.990)	0.0262	**0.7413**	0.8582	0.9693
	rs2289163	*RORA*	chr15:60,590,769: A:C	0.905 (0.812–1.010)	0.0742	0.891 (0.796–0.997)	0.0433	**0.7982**	0.8930	0.9775
	*recessive*									
	rs8033552	*RORA*	chr15:60,551,386: G:A	1.195 (1.004–1.422)	0.0452	1.250 (1.044–1.496)	0.0151	**0.7403**	0.8575	0.9691
	rs10857561	*MAPK8*	chr10:48,400,595: G:A	1.115 (1.011–1.229)	0.0294	1.112 (1.005–1.229)	0.0391	**0.7802**	0.8822	0.9750
	rs13515	*MAPK1*	chr22:21,761,597: G:A	0.828 (0.687–0.997)	0.0468	0.795 (0.656–0.964)	0.0195	**0.7741**	0.8786	0.9742

*BFDP* Bayesian false-discovery probability, *OR* Odds ratio, *CI* Confidence interval

Chromosome and position (GRCh38.p13): For example, chr9:91,426,574: A:G indicates chromosome 9, base pair location 91,426,574, referent allele A, variant allele G

^a^Adjusted for study, reference age and eight principal components

^b^Adjusted for study, reference age, parity, breast feeding, smoking status, current intake of estrogen-progesterone MHT, and eight principal components

^*^Supplementary table S4 provides an overview of all models for the stratified analysis by breast cancer hormone receptor status

### Interaction analysis

The 20 most important interaction terms each from three different starting seeds for the logicFS algorithm resulted in 53 unique interactions terms (Suppl. table S5). The adjusted logistic regression models for these terms yielded ten interaction terms with a p-value < 0.05 (Suppl. table S6), hence suitable for BFDP calculation.

With a prior probability for an effect of 0.01, we found three noteworthy interaction terms in the adjusted models (BFDP < 0.8, Table 4): rs10857561_*R*_ ∧ !rs1347069_*D*_ (OR = 1.15, 95% CI 1.05–1.25), rs10857561_*R*_, (OR = 1.11, 95% CI 1.04–1.18), and rs1386483_*R*_ ∧ rs1473473_*D*_ ∧ rs3729931_*D*_ (OR = 1.20, 95% CI 1.09–1.32). With increased priors of 0.05 and 0.1, in total six and eight interaction terms reached noteworthiness, respectively. All noteworthy interactions (all priors) included at least one SNP that showed noteworthy associations, respectively, in the main effect analysis (*TPH2*: rs1386489, rs1473473, rs7299582; *MAPK8*: rs10857561, rs7075976).

**Table 4:** Breast-cancer risk associations and Bayesian false-discovery probability (BFDP, bold text indicates BFDP < 0.8) for interactions/main effects with *p* value < 0.05 in adjusted logistic regression models*, sorted by OR^b^

Interaction/main effect^†^	Nearest Genes	OR^a^** (95% CI)	OR^b^** (95% CI)	BFDP for OR^b^ Prior probability for effect (β ≠ 0)		
				**0.1**	**0.05**	**0.01**
rs1386483_*R*_ ∧ rs1473473_*D*_ ∧ rs3729931_*D*_	*TPH2, TPH2, RAF1*	1.163 (1.064–1.270)	1.201 (1.090–1.323)	**0.082**	**0.159**	**0.495**
rs10857561_*R*_ ∧ !rs1347069_*D*_	*MAPK8, MAP2K1*	1.156 (1.070–1.249)	1.147 (1.053–1.248)	**0.247**	**0.409**	**0.783**
rs1386483_*R*_ ∧ rs1473473_*D*_ ∧ !rs2269457_*D*_	*TPH2, TPH2, NR1D1*	1.095 (1.001–1.197)	1.113 (1.009–1.227)	**0.759**	0.869	0.972
rs10857561_*R*_	*MAPK8*	1.120 (1.056–1.188)	1.109 (1.040–1.183)	**0.253**	**0.417**	**0.788**
rs1386489_*D*_ ∧ !rs3828057_*R*_	*TPH2, RORC*	1.064 (1.015–1.114)	1.084 (1.030–1.141)	**0.288**	**0.461**	0.817
rs1473473_*D*_	*TPH2*	1.050 (1.007–1.094)	1.067 (1.019–1.116)	**0.463**	**0.645**	0.904
rs7075976_*R*_	*MAPK8*	1.068 (1.017–1.121)	1.066 (1.010–1.124)	**0.710**	0.838	0.964
rs7299582_*D*_	*TPH2*	1.038 (0.994–1.084)	1.065 (1.015–1.116)	**0.580**	**0.745**	0.938
!rs2171363_*R*_ ∧ !rs7026487_*D*_ ∧ !rs9610375_*R*_	*TPH2, RORB, MAPK1*	1.054 (1.010–1.101)	1.051 (1.002–1.103)	0.840	0.917	0.983
!rs12941497_*D*_	*NR1D1*	1.040 (1.000–1.081)	1.044 (1.000–1.089)	0.857	0.927	0.985

*BFDP* Bayesian false-discovery probability, *OR* Odds ratio, *CI* Confidence interval

^†^∧ indicates Boolean "AND" conjunction; ! indicates Boolean "NOT" operator; _D_ and _R_ indicate SNP coding according to the dominant or recessive genetic model, respectively

^a^Adjusted for study, reference age, and eight principal components

^b^Adjusted for study, reference age, parity, breast feeding, smoking status, current intake of estrogen-progesterone MHT, and eight principal components

*Supplementary tables S5 and S6 provide overviews of the 20 most important interactions and their logistic regression models, respectively

**ORs and 95% CIs differ slightly from Suppl. table S3 results because all observations with missing data for any SNP were removed

## Discussion

This hypothesis-based breast-cancer association study focused on the putative role of modulators of the pineal gland hormone melatonin and their potential influence on breast-cancer risk. In line with the light-at-night hypothesis, according to which altered light-induced nocturnal melatonin production and signaling increases the risk of breast cancer [3], our findings point to a cooperative role of genetic variations that may modulate serotonergic brain networks and/or the signaling of melatonin within the context of breast-cancer susceptibility.

The strongest observed risk effects were driven by various interactions of polymorphisms at *TPH2* and *MAPK* genes (*MAPK8*, *MAP2K1, RAF1*). The triple interaction of *TPH2* rs1386483 and rs1473473 as well as *RAF1* rs3729931 increased breast-cancer risk by 20%, a dual interaction of *MAPK8* rs10857561 and *MAP2K1* rs1347069 by 15%, and *MAPK8* rs10857561 alone by 11% (all observed at a prior probability of 0.01). In most instances, risk effects were evident at the individual SNP level both, in main and stratified risk analyses by hormone-receptor status. To the best of our knowledge and based on the combined iCOGs/Oncoarray meta-analysis of the BCAC cohort [20] as well as the Catalogue of Curated Breast Cancer Genes [28], these breast-cancer risk associations are newly described. Yet, some *TPH2* polymorphisms have been reported in the literature within the context of psychiatric disorder related endpoints such as antidepressant response and GABA concentration, conditions in which effects of serotonin are underlying biological mechanisms [29, 30].

The involvement of *TPH2* as a putative breast cancer susceptibility locus is plausible, as the enzyme is exclusively expressed in neuronal cells of the central nervous system where it catalyzes the rate limiting step in serotonin (5-HT) biosynthesis, the chemical precursor of melatonin [31]. Its pertinent role in females has been inferred from expression studies of post mortem brain tissues in which female thalamic and hypothalamic brain showed higher *TPH2* mRNA expression levels compared to male counterparts [32], thereby highlighting the critical role of an intact serotonergic pathway for female neurohormone/neurotransmitter production. In general, TPH2 is present in various brain regions with particular abundance in the major central serotonergic neuronal networks that localize to median and dorsal raphe nuclei in the brainstem known to participate in basal functions such as temperature regulation, feeding and energy balance, as well as mood and sleep [33, 34]. The synthesis and periodical secretion of serotonin from these brain stem regions and neuronal terminal fields are regulated at the level of *TPH2* gene expression that was shown to be under the circadian control of melatonin, estrogen, and corticoids in rodents and primates [34, 35]. This underscores the various feedback mechanisms to the serotonergic system that are involved in the entrainment of the hypothalamic SCN [7, 36, 37]. To a smaller extent, *TPH2* is also rhythmically expressed in ocular tissues with rhythmical release of melatonin, the levels of which are highest in darkness and lowest in the light [8, 38]. Intrinsic photosensitive retinal ganglion cells by virtue of their concurrent rhythmic melatonin-receptor expression may therefore contribute to the output signal of the retino-thalamic tract to the SCN [39]. Hence, our findings of an association between *TPH2* variants and increased breast-cancer risk are well in line with the notion that such variants impact on serotonin formation, thereby disrupting the SCN pacemaker circuitry feedback. In particular, the observed increased breast-cancer risk in *TPH2* variant carriers may result from modified brain melatonin levels due to a dysfunctional SCN (together with reduced melatonin levels in the retina) that upon light-at-night periods may reduce pineal melatonin secretion.

If the disruption of pineal gland melatonin biosynthesis during extended light-at-night periods affects nocturnal serotonin levels and transmission to the hypothalamic nuclei SCN and PVN [7, 40], we need to consider further that these nuclei also make up major parts of the hypothalamic-pituitary–gonadal (HPG) axis. In the HPG axis, serotonin modulates the hypothalamic secretion of the gonadotropin releasing hormone (GnRH) as well as the pituitary luteinizing and follicle stimulating hormones (LH, FSH), and prolactin that stimulate the production of sex hormones in peripheral target organs such as the ovaries or testes [41]. Similar to melatonin, prolactin secretion may be driven by the central circadian pacemaker located in the SCN of the hypothalamus [42], and our observed *TPH2* variant-associated increased breast-cancer risk may in addition relate to local serotonergic effects accountable for increased prolactin production in the anterior pituitary gland. Of note, circulating prolactin is an established breast-cancer risk factor that has been confirmed in a series of analyses from the prospective Nurses’ Health Study, particularly with respect to ER-positive breast cancer [43], and a large analysis from the prospective EPIC cohort [44] with emphasis on users of hormone replacement therapy. This is underpinned by a vast body of evidence from animal and in vitro studies. Together with estradiol and progesterone, it exerts effects on normal epithelial cell expansion, ductal side branching of the breast during puberty, and formation of lobuloalveolar structures during pregnancy [45]. As prolactin and progesterone have synergic roles to induce cell growth and proliferation during adult gland maturation and alveologenesis of the breast terminal duct-lobular units, the site of origin for most breast cancers, a crosstalk between progesterone, prolactin and receptor signaling pathways may not only be relevant in normal, but also malignant breast cells [46].

*MAPK8* (JNK, c-Jun N-terminal kinase) in our study has been associated with breast-cancer risk at rs10857561, both in the individual main and hormone-receptor positive (ER + and PR +) stratified analyses, as well as in the SNP-SNP interaction analyses. While all three canonical MAPK pathways (ERK MAPK, p38 MAPK, and JNK MAPK) serve as input to the circadian clock in distinct ways [9], JNK in particular has been shown to be essential for the normal oscillation of the mammalian circadian clock and its photic regulation. The JNK-imparted transmission of light signals to the BMAL1-CLOCK complex controls oscillation speed and phase response of the master clock [9].

Aside from their role as master clock regulators via SCN or peripheral tissue, MAPK genes play a role in cellular processes like proliferation and cell death [47]. It is therefore not surprising that they were associated with the development of cancer at many sites [48]. While rs10857561 in *MAPK8* showed noteworthy risk estimates for breast cancer in general as well as ER-positive and PR-positive subgroups in our analyses, this SNP has been previously shown to be associated with rectal cancer once more highlighting the role in carcinogenesis [49].

A major strength of this study is the large number of study participants (22,992 cases and 21,413 controls) retrieved from population-based case–control studies with defined reference age (age at diagnosis for cases, age at interview for controls) and availability of comprehensive epidemiological and tumor immunohistochemistry as well as genotype data. This allowed us to calculate precise SNP-specific OR main effect and interaction estimates. Our hypothesis-driven approach of a putative role of polymorphic regulators or signaling mediators of melatonin in breast-cancer risk limited the number of potentially detectable false-positive associations. Moreover, we used the BFDP to measure the noteworthiness of our effect estimates and to account for false-positive results via multiple testing. Furthermore, our interaction analysis based on logic regression models enabled us to model the effects of complex interaction scenarios considering the multivariate structure of SNP interplay.

In spite of the large study size, limitations of the study include a high number of missing values in included confounders. We used the category ‘unknown’ for categorical variables in these participants to maintain the remaining information. Moreover, the sample size in subgroup analyses was reduced, for example, ER status was missing for 21% of samples and PR status for 36% of samples. However, missingness is likely to be random with respect to genotypes. There was also minor heterogeneity in definition of stage, grade, and cut-off levels for ER and PR across studies. The tumor subtypes were strictly defined by immunohistochemical markers as other data, such as intrinsic subtypes from expression profiles nowadays used for subtype definition, are not available in large-scale epidemiological studies. Finally, our interpretations strictly rely on functional and physiological data reported in the literature and include in vitro, animal in vivo as well as post mortem findings.

Our newly identified breast-cancer risk associations justify continued research into the relationship between breast-cancer risk and putative modulators of the intricate network of rhythmic circadian regulators such as melatonin and serotonin upon photic stimulation at night. The observed interactions between genetic variants of *TPH2* and *MAPK8* highlight their cooperation as putative breast-cancer risk modulators and call upon the comprehensive scrutiny of the circadian clock system and its input and output effectors in large breast-cancer cohorts. This research holds the potential to reveal new insights into the breast-cancer risk of women exposed to light-at-night which is particularly relevant for female night shift workers.

### Supplementary Information

Below is the link to the electronic supplementary material.

Supplementary file1 (XLSX 20 KB)

Supplementary file2 (XLSX 30 KB)

Supplementary file3 (XLSX 43 KB)

Supplementary file4 (XLSX 130 KB)

Supplementary file5 (XLSX 15 KB)

Supplementary file6 (XLSX 21 KB)

## Data Availability

The datasets analyzed in the current study are available via the BCAC Data Access Coordinating Committee (bcac@medschl.cam.ac.uk) upon reasonable request. Summary-level genotype data are available via http://bcac.ccge.medschl.cam.ac.uk and in supplementary table S2. Individual-level data are available via the BCAC Data Access Coordinating Committee (bcac@medschl.cam.ac.uk).
